# Recent Advances in Heterogeneous Hydroformylation at Metal–Oxide Interfaces

**DOI:** 10.3390/molecules30204078

**Published:** 2025-10-14

**Authors:** Maxwell Gillum, Gallage K. P. A. Ariyaratne, Charbel Tawny, Paul Alimenti, Kyle Krawczykowski, Erik Polik, Mausumi Mahapatra

**Affiliations:** Department of Chemistry & Biochemistry, Loyola University Chicago, Chicago, IL 60660, USA; mgillum@luc.edu (M.G.);

**Keywords:** hydroformylation reaction, metal/oxide interface, nanoparticles, single atom catalysis

## Abstract

This article reviews recent advances in heterogeneous hydroformylation, with a particular focus on rhodium-based catalysts supported on oxide surfaces. The hydroformylation reaction is a vital industrial process for producing aldehydes from petrochemicals. This reaction involves the addition of carbon monoxide (CO) and hydrogen (H_2_) to alkenes, resulting in the formation of aldehydes. Aldehydes serve as essential building blocks for various downstream products in the chemical industry, including alcohols, esters, and amines. Although homogeneous catalysts such as rhodium complexes coordinated with phosphorus-based ligands (e.g., [RhCl(PPh_3_)_3_]) are highly active and selective, their separation and recovery remain significant challenges. This has fueled growing interest in the development of heterogeneous catalysts, which offer advantages in terms of sustainability, reusability, and catalyst recovery. This review highlights recent progress in the design of heterogeneous hydroformylation catalysts, with emphasis on rhodium-based systems on oxide supports. Key challenges and emerging strategies for enhancing catalytic performance and stability are also discussed.

## 1. Introduction

**Figure 1:**
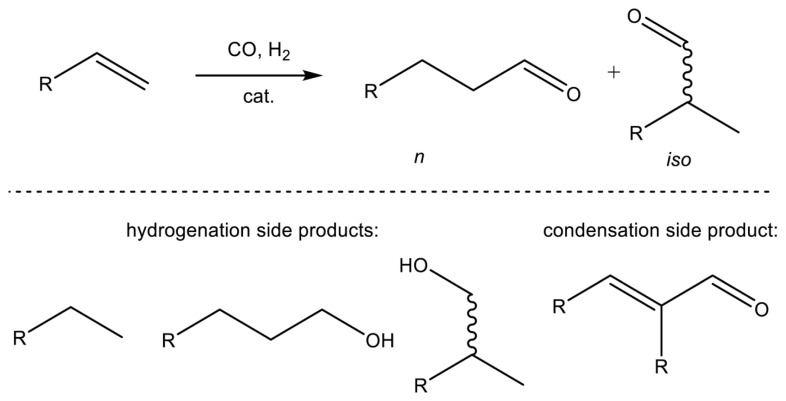
Reaction schematic of hydroformylation of an olefin leading to the formation of n- and iso- aldehyde and the formation of hydrogenation and aldol condensation side products. Image reproduced from reference [3].

Hydroformylation is an important industrial process used to convert alkenes into aldehydes through the addition of carbon monoxide (CO) and hydrogen (H_2_). The resulting aldehydes are key intermediates in the synthesis of a wide range of downstream chemicals, including alcohols, esters, and amines [1]. Controlling product selectivity in hydroformylation reactions poses several challenges [1,2,3]. For example, hydroformylation of alkenes other than ethylene often yields both linear and branched aldehydes, necessitating precise control of reaction conditions to favor the desired product. Additionally, dominant side reactions, such as hydrogenation of olefins to their corresponding alkanes at higher temperatures, can further impact selectivity (Figure 1). Achieving maximum selectivity for hydroformylation reactions thus demands careful catalyst design and control of reaction parameters such as temperature and pressure.

This reaction has been studied extensively in the homogeneous catalysis field, where the most active catalysts are reported to be unmodified or ligand-modified Rh complexes such as [RhCl(PPh_3_)_3_]. Several other transition metals also have been investigated for this reaction, and the order of activity for unmodified metals in homogeneous catalysis generally follows Rh > Co > Ir > Ru > Os > Pt > Pd > Fe > Ni [2,4]. Indeed, while homogeneous catalysts like [RhCl(PPh_3_)_3_] offer high activity and selectivity in hydroformylation reactions, these systems are typically plagued by challenges related to catalyst separation from the reaction mixture and leaching of the phosphine linker during the reaction, leading to the deactivation of the catalysts. To address these issues, increasing attention has been directed toward the design of heterogeneous catalysts and processes for hydroformylation reactions [3].

The support is a key portion of heterogeneous catalysis and a variety of solid supports have been explored for hydroformylation reactions, including metal–organic frameworks (MOFs), [5,6] oxides, [7,8,9,10,11,12] and carbon-based materials to selectively disperse and activate metal catalysts [13,14,15,16]. This review focuses on Rh-based active sites supported on various oxides.

**Figure 2:**
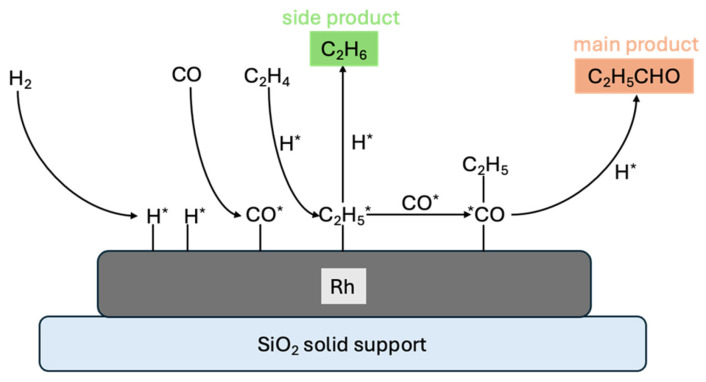
Schematic representation of reaction pathway for hydroformylation of ethylene on Rh/SiO_2_ catalyst material as proposed by Chuang and Pien [3]. * Denotes surface-bound species.

The stabilization of metal particles on oxide support is longstanding in the design of various catalytic processes and reactions due to the versatile nature of the oxide supports and the intricate nature of the metal/oxide interface, which has unique properties which allow it to catalyze reactions with high activity and selectivity [17,18,19]. In the context of hydroformylation, rhodium (Rh) nanoparticles supported on silicon dioxide (SiO_2_) represent a benchmark system for heterogenizing the reaction. This approach was first pioneered by Chuang and Pien [20,21]. Since the publication of their foundational work, extensive research has focused on improving catalytic activity and product selectivity by exploring a variety of oxide supports. These developments have been summarized in several comprehensive review articles [1,3,12]. Based on these studies, the widely accepted reaction mechanism for ethylene hydroformylation over Rh/SiO_2_ catalysts (illustrated in Figure 2) involves the following key steps: (1) Hydrogenation of C_2_H_4_ with an adsorbed *H species, leading to the formation of a hydrogenated alkyl species, (2) CO insertion into the C-Rh bond of an adsorbed alkyl species, and (3) hydrogenation of the adsorbed alkyl species, resulting in aldehyde formation. Among these steps, CO insertion is reported to be the rate-determining step of the reaction [3].

As a result, most earlier studies focused on understanding the interaction of CO with Rh and how the oxidation state of Rh (Rh^+^ vs. Rh^0^) affects CO bonding and hydroformylation performance [22,23]. It has been reported that Rh^+^ tends to favor linear CO bonding, while Rh^0^ promotes bridging CO bonding—both of which have direct implications for catalytic activity. Earlier researchers proposed that Rh in a zero oxidation state (Rh^0^) is the active catalyst for hydroformylation [22], whereas other studies indicate that Rh with +1 oxidation state (Rh^+1^) is the active catalyst particularly when supported on SiO_2_ [11].

The CO–Rh interaction was thoroughly investigated in an earlier study by McClure et al., who examined the surface chemistry of ethylene hydroformylation on an Rh/SiO_2_ model surface and established a clear structure–activity relationship [11]. This work will be discussed in more detail in Section 2, which focuses on model studies of hydroformylation. To date, it remains the only study examining hydroformylation on well-defined model surfaces by experimental investigation.

The primary focus of this paper, however, is on recent advances over the past decade, during which research has increasingly emphasized the design of single atoms, bifunctional sites, and nanoclusters supported on oxides. Many of these studies aim to develop single-atom catalysts as a means of bridging the gap between homogeneous and heterogeneous catalysis. However, the synthesis and stabilization of single atoms remain major challenges due to their inherent tendency to agglomerate into clusters or nanoparticles in order to minimize surface free energy. Consequently, a significant body of literature examines the combined effects of single atoms and clusters on hydroformylation activity and selectivity [7,9,24]. These aspects will be discussed in detail in Section 3 and Section 4. Section 5 presents concluding remarks and outlines future directions for improving catalyst design and catalytic performance.

## 2. Surface Chemistry of Hydroformylation: Model Studies

**Figure 3:**
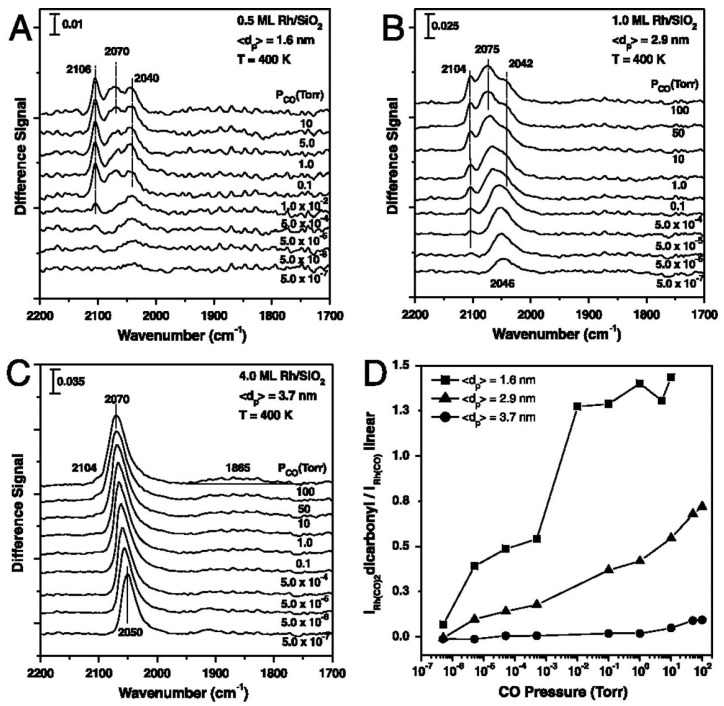
Polarization modulation reflection absorption infrared spectra of SiO_2_-supported Rh nanoparticles of initial size (**A**) 1.6 (**B**) 2.9 and (**C**) 3.7 nm during exposure to increasing CO pressures at 400 K. Spectra obtained sequentially from low to high CO pressures; scale bar denotes relative scale between figures. (**D**) Ratio of the Rh(CO)_2_ *ν_CO_* stretching intensity to the linear bound *ν_CO_* stretching intensity (I_Rh_ (CO)_2, sym_/I_Rh_ (CO)_linear_) as a function of pressure (Torr) from the data of (**A**–**C**). Image reproduced from reference [11].

Surface chemistry studies on well-defined model systems often provide critical insights into the nature of catalytic materials, the nature of active sites, and the mechanistic details of reaction pathways at the atomic scale. Although catalytic reactions and processes are inherently complex, surface science approaches—particularly those involving well-defined single-crystal surfaces of specific facets and experiments conducted under ultrahigh vacuum (UHV) conditions—allow for significant simplification. This controlled environment enables the elucidation of reaction mechanisms with atomic-scale precision. McClure et al. studied ethylene hydroformylation on model Rh/SiO_2_ surfaces which provided a comprehensive study of the structure–activity behavior [11]. The findings of their study indicated that the particle size of Rh plays a critical role in influencing the CO insertion step, thereby exerting control over the catalytic activity for the hydroformylation reaction. Their study indicated that CO insertion activity reached an optimum for Rh particle sizes around 2.5 nm. Overall, their study suggests that the particle size affects the CO insertion step by two factors: (1) promotion of the CO insertion on low coordinated Rh surface sites, and (2) small particles (less than 2.5 nm) lead to a decrease in activity, probably due to the formation of dispersed Rh carbonyl hydride species. Hence, the optimization of Rh particle size is crucial, ensuring it is small enough to create a significant number of low coordination sites for CO insertion. However, it should not be excessively small to impede the dispersion induced by CO and H_2_. The authors used polarization modulation reflection absorption infrared spectroscopy (PM-IRAS) to probe CO-Rh interaction as illustrated below in Figure 3.

PM-IRAS spectra were taken of SiO_2_-supported 1.6, 2.9, and 3.7 nm Rh nanoparticles during low-to-elevated pressures of CO exposures (surface temperature = 400 K). For measurements taken at CO pressures below 5 × 10^−6^ Torr, a singular vibrational feature was observed which is consistent with linear and bridging-bound CO on Rh particles (Figure 3A–C). As the pressure of CO is increased above 5 × 10^−6^, the onset of two additional features at 2100 cm^−1^ and 2040 cm^−1^ were observed. These features are consistent with the symmetric (2090–2100 cm^−1^) and asymmetric (2020–2035 cm^−1^) stretching modes of gem-dicarbonyl Rh(CO)_2_. As stated by McClure et al., the appearance of these new vibrational features indicates a disruption and dispersion of the Rh nanoparticles by elevated CO pressures. The ratio of intensities for the gem-dicarbonyl and linear stretching frequencies observed in Figure 3A–C indicate that this disruption is attenuated on larger Rh particles, which is consistent with previous studies of TiO_2_ [25] and Al_2_O_3_ [26] supported Rh particles.

**Figure 4:**
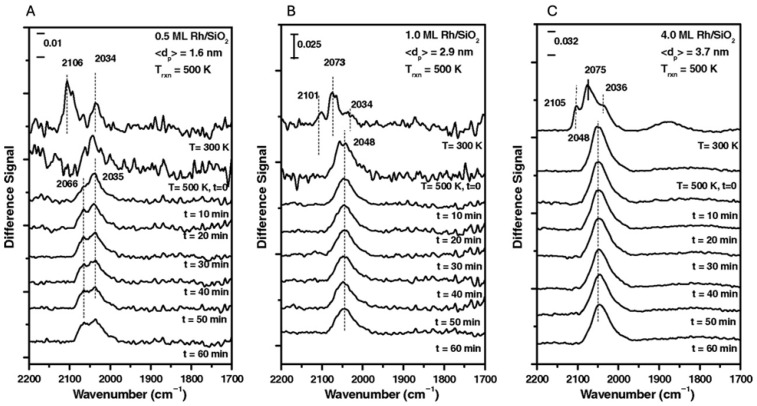
Polarization modulation reflection absorption infrared spectra of SiO_2_ supported (**A**) 1.6 nm (**B**) 2.9 nm and (**C**) 3.7 nm Rh nanoparticles under ethylene hydroformylation reaction conditions (50 Torr CO, 50 Torr C_2_H_4_, and 400 Torr H_2_). The sample was then heated to T = 500 K and another rapid IR spectrum was obtained, with more spectra obtained every 10 min as the sample was held at the reaction temperature for one hour. The image is reproduced from reference [11].

When these Rh particle sizes were studied under C_2_H_4_/CO/H_2_ reaction conditions (T = 500 K), as illustrated by the PM-IRAS spectra in Figure 4, particle size again played a critical role in determining the nature of surface-bound species. The reactants were introduced at 300 K and the PM-IRAS spectra were taken every 10 min at a reaction temperature of 500 K. For initial particle sizes of 2.9 nm and larger (Figure 4B,C), a single vibrational mode corresponding to linearly bound CO was observed at the reaction temperature. In contrast, for smaller particles (1.6 nm, Figure 4A), stretching frequencies consistent with both linearly bound CO (~2066 cm^−1^) and an Rh carbonyl hydride species, Rh(CO)H (~2035 cm^−1^), were detected. The assignment of Rh(CO)H peak is based on previous literature [27,28]. Interestingly, McClure et al. proposed that the formation of the Rh(CO)H species may be linked to the lower turnover frequencies (TOFs) observed at smaller average particle sizes. Furthermore, the presence of Rh(CO)H may influence selectivity, shifting selectivity from propanal (CH_3_CH_2_CHO) toward ethane (C_2_H_6_). Although 1.6 nm Rh particles exhibited low activity for propanal formation, they displayed significantly higher TOFs for ethane production compared to larger particles.

These findings suggest that, for Rh particles smaller than 2.9 nm, changes in CO binding behavior and particle dispersion can lead to the formation of Rh(CO)H species, which may in turn suppress hydroformylation activity while promoting hydrogenation to ethane.

While this study remains one of the most comprehensive efforts to evaluate hydroformylation performance and establish a structure–performance relationship on a model Rh/SiO_2_ surface, there is evidence in the literature of experimental studies that have focused on probing individual components of the reaction, such as the interactions of CO, C_2_H_4_, and H_2_ with Rh supported on other oxides which might provide crucial information in the design of novel catalysts [29,30,31].

Computational studies play a crucial role in understanding atomistic details of catalytic processes and in guiding the rational design of improved catalysts. Some of the structural sensitivities discussed above were examined in a recent study by Ghoshal et al., who conducted a comprehensive evaluation of various metal catalysts supported on SiO_2_ for ethylene hydroformylation [32]. By integrating experimental data with theoretical modeling, the study investigated how structural features of metal catalysts influence their performance in this reaction [32]. The metals examined included Rh, Pt, Ir, Ni, Au, Ag, Pd, and Cu, with rhodium emerging as the most selective for producing propanal (C_2_H_5_CHO) via the C–C coupling step in the experimental results.

To gain mechanistic insights into these selectivity trends among these metals, the authors employed density functional theory (DFT) calculations in conjunction with microkinetic modeling. Their study highlights the critical importance of using an appropriate computational model to accurately capture experimental observations. Notably, traditional surface models, where low-index facets of a metal surface are typically used to represent metal nanoclusters, failed to reproduce the experimentally observed activity and selectivity trends. In contrast, cluster models provided a more realistic representation of the active catalytic sites and were successful in describing the catalytic behavior observed in this system, as detailed below.

**Figure 5:**
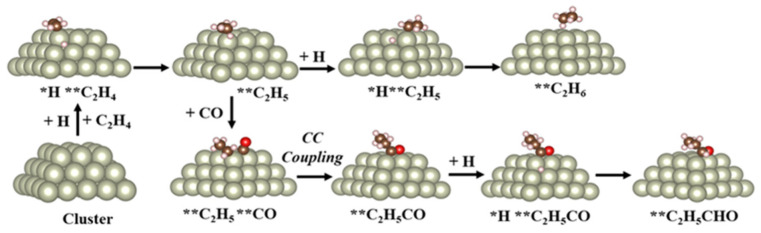
Schematic pathways of ethylene hydrogenation and hydroformylation on metal nanoclusters, denoting the binding of reactants and intermediates to corner and edge sites with ** and *, respectively. The elements are assigned with the following colors: hydrogen, white; carbon, brown; oxygen, red; metal cluster, light green. The image is reproduced from reference [32].

Using the cluster model, the authors have shown that Rh nanoclusters possess the lowest energy barrier for the C–C coupling step, corroborating the high aldehyde selectivity observed experimentally. The DFT results further revealed that planar Rh surfaces primarily drive hydrogenation, favoring the formation of ethane (C_2_H_6_), whereas undercoordinated sites at the edges and corners of the clusters selectively facilitate C–C coupling. Species bound to the edge and corner site are denoted by * and **, respectively. The authors have surmised that C_2_H_4_ first binds to a corner site (**) of the metal cluster. The **C_2_H_4_ then undergoes hydrogenation through interaction with dissociatively adsorbed *H atoms, forming a **C_2_H_5_ intermediate. There are then two separate reaction pathways available. One pathway allows the **C_2_H_5_ intermediate to proceed through further hydrogenation, producing ethane (C_2_H_6_) gas. The more preferential pathway, for use in hydroformylation chemistry, is that the intermediate species undergoes a two-step hydroformylation pathway in which the **C_2_H_5_ couples with CO to form the corner-bound **C_2_H_5_CO intermediate, which is then hydrogenated to produce propanal (*C_2_H_5_CHO). The reaction mechanism is shown below in Figure 5 [32].

What is interesting about the simulations performed by Goshal et al. is that the selectivity for either C_2_H_6_ or C_2_H_5_CHO can be linked to the adsorption strength of CO to the metal cluster [32]. Among the four metal clusters used in these simulations (Rh, Pt, Pd, and Ir), CO most strongly bound to the Ir clusters (−2.14 eV) compared to Pd clusters, which had the lowest CO binding energy of −1.87 eV. It was determined that the Rh clusters, which had a more intermediate CO binding strength of −1.98 eV, was the most selective catalyst for propanal formation. This is thought to be partially due to CO binding too strongly to the Ir (−2.14 eV) and Pt (−2.04 eV) clusters, which increases the activation energy of C_2_H_5_ and CO coupling, thereby reducing selectivity for C_2_H_5_CHO production and increasing selectivity towards C_2_H_6_ production. Conversely, Pd clusters may bind CO too weakly due to the formation of PdH_x_ in a H_2_ atmosphere [33]. These insights contribute significantly to the understanding of catalytic performance at the nanoscale, emphasizing that not only the choice of metal but also the control over nanoparticle morphology is critical. By optimizing Rh particle shape and size to increase the density of reactive edge and corner sites, catalytic efficiency and selectivity in hydroformylation can be effectively improved. These findings are consistent with the earlier work of McClure et al., who proposed that low-coordination sites on Rh nanoparticles play a crucial role in the hydroformylation process by facilitating the CO insertion step [11].

## 3. Rh Single Atoms and Clusters on Oxide Support

Recent investigations have mostly focused on the stabilization of Rh single-atom catalysts on various oxide supports. Single-atom catalysis (SAC) is the new frontier in catalysis science owing to their unique electronic and chemical properties that lead to catalyzing reactions with high activity and selectivity [34,35,36,37,38,39,40,41,42]. Furthermore, the high atom efficiency of SAC systems makes them a highly cost-effective approach. Single-atom catalysts also serve as a bridge between homogeneous and heterogeneous catalysis. The major challenge for the design of the proposed active sites is related to the thermodynamic instability of the single-site catalysts. It is well known that the single atoms agglomerate to form clusters and nanoparticles during the synthesis process to minimize the surface free energy. In addition, it is common for single atoms to sinter to nanoparticles under reaction conditions. Despite the existing hardships, there are growing investigations in this field and there are a large number of studies which focus on the synthesis of such single-atom catalysts on various oxide surfaces and their stabilization under reaction conditions. In the context of hydroformylation, several strategies have been explored in SAC research. These include controlling metal loading and optimizing the synthesis procedure to prevent atom clustering [9,24,43], investigating the influence of different oxide supports and defects [7,12,24], and studying metal–support interactions [43,44]. These factors play a critical role in determining hydroformylation performance. Selected examples from the literature are discussed below to illustrate these effects.

### 3.1. Effect of Various Oxide Support and Defects

The stabilization of Rh single atoms has been extensively studied on various oxide supports to understand the influence of the support on single-atom stabilization and hydroformylation performance [7,9,12,24,44]. Most studies in this field employ a combination of spectroscopic (e.g., near edge X-ray absorption fine structure (NEXAFS), X-ray absorption near edge structure (XANES), X-ray photoelectron spectroscopy (XPS)) and microscopic (e.g., scanning transmission electron microscopy (STEM), scanning electron microscopy (SEM), transmission electron microscopy (TEM)) techniques to determine the relative proportions of Rh single atoms and clusters present on the surface. Microscopic techniques provide direct visualization of these species, while spectroscopic methods such as X-ray absorption near-edge spectroscopy (XANES) and extended X-ray absorption fine structure (EXAFS) are used to investigate the electronic and local chemical environments characteristic of single atoms and clusters. Another common approach involves using infrared (IR) spectroscopy to probe the CO–Rh interaction as shown above in Section 2, Figure 3 and Figure 4. Rh single atoms typically exhibit a distinct IR peak corresponding to gem-dicarbonyl species, whereas Rh clusters display IR signals typically associated with linearly bonded and bridge-bonded CO. The majority of publications report catalytic performance metrics, including activity and selectivity for aldehyde production, catalyst stability over time, and comparisons with other catalysts reported in the literature. Most of these studies focus on powder or nanoparticulate catalysts, with minimal experimental investigation of single-crystal surfaces. However, a significant number of studies incorporate density functional theory (DFT) calculations, often performed on well-defined surfaces to correlate with the experimental findings. These typically use specific facets of single-crystal supports to model reaction energetics and pathways. A few representative examples are provided below.

**Figure 6:**
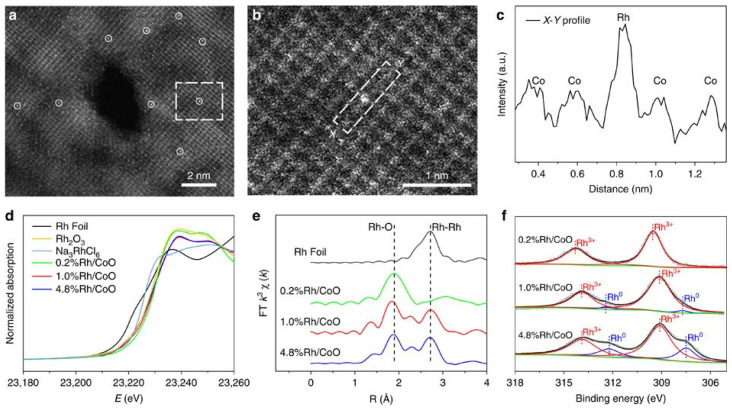
Structural characterization of Rh/CoO with different Rh loadings. (**a**) High-angle annular dark-field scanning transmission electron microscopy (HAADF-STEM) image of 0.2% Rh/CoO single-atom structures. Rh single atoms marked in white circles. (**b**) Magnified HAADF-STEM image of 0.2% Rh/CoO single-atom structures corresponding to the area in the white square in (**a**). (**c**) Intensity profile along the line X-Y in the magnified HAADF-STEM in (**b**). (**d**) Rh K-edge X-ray absorption near edge structure profiles for Rh/CoO with different Rh loadings. (**e**) Rh K-edge extended X-ray absorption fine structure spectra in R space for Rh/CoO with 0.2%, 1.0%, and 4.8% Rh loadings. (**f**) X-ray photoelectron spectra of Rh/CoO with 0.2%, 1.0%, and 4.8% Rh loadings. Image reproduced from reference [9].

Zeng et al. investigated propene hydroformylation using Rh single-atom catalysts supported on CoO nanosheets [9]. The surface of the catalysts was characterized using high-angle annular dark-field scanning transmission electron microscopy (HAADF-STEM, JEOL ARM-200 F field-emission transmission electron microscope, Tokyo, Japan), X-ray absorption near-edge spectroscopy (XANES), extended X-ray absorption fine structure (EXAFS), and X-ray photoelectron spectroscopy (XPS), as presented in Figure 6.

At a low Rh loading of 0.2 wt%, isolated Rh atoms were observed to substitute surface Co atoms. In contrast, higher Rh loadings of 1.0 wt% and 4.8 wt% led to the formation of Rh clusters, as evidenced by the HAADF-STEM images and corresponding line profile measurements (Figure 6a–c). To probe the electronic structure and local chemical environment, XANES and EXAFS measurements were performed. The Rh K-edge XANES spectra (Figure 6d) revealed that Rh species in the 0.2%Rh/CoO catalyst existed in a more oxidized state than those in the 1.0% and 4.8% samples. This conclusion was based on the higher absorption edge energy and increased white-line intensity. In the EXAFS R-space spectra (Figure 6e), the 0.2%Rh/CoO catalyst showed a strong peak near 2.0 Å, corresponding to Rh–O coordination with a coordination number (CN) of 5.8. Peaks associated with Rh–Rh or Rh–Cl interactions at longer distances (>2.0 Å) were not detected, suggesting that Rh atoms were uniformly dispersed without forming aggregates. With Rh loadings raised to 1.0 wt% and 4.8 wt%, a new peak emerged around 2.7 Å, signaling the onset of Rh–Rh metallic bond formation (Figure 6e). The coordination number for Rh–O also declined to 4.6 and 4.1 in the 1.0% and 4.8% samples, respectively. To further examine the electronic states of Rh, XPS analysis was carried out. As shown in Figure 6f, the Rh 3d_5_/_2_ binding energy for the 0.2%Rh/CoO catalyst was measured at 309.5 eV, indicating the presence of Rh^3+^ single atoms. In the 1.0% and 4.8%Rh/CoO samples, this peak shifted slightly to 309.2 eV and 309.1 eV, respectively. New peaks also appeared at 307.7 eV and 307.5 eV, which were attributed to metallic Rh, confirming the formation of Rh clusters at higher loadings.

**Figure 7:**
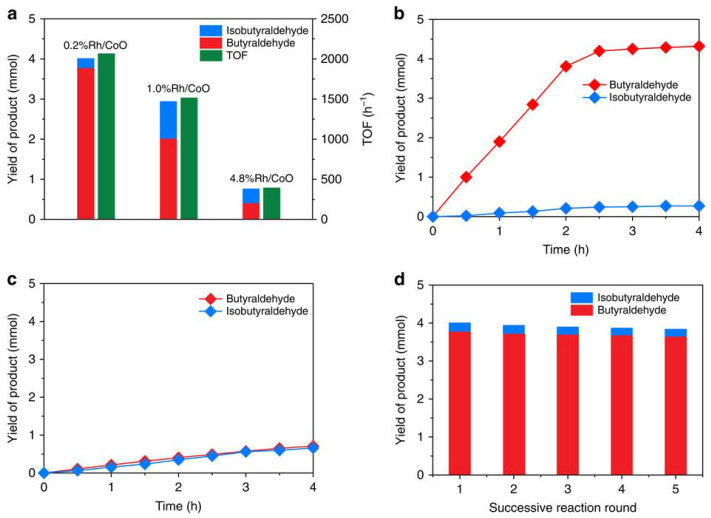
Catalytic performance in the hydroformylation of propylene. (**a**) Turnover frequencies (TOFs) and product yields of 0.2% Rh/CoO, 1.0% Rh/CoO, and 4.8% Rh/CoO in the hydroformylation of propene at 100 °C after 2 h. (**b**,**c**) Time courses of the hydroformylation of propene over 0.2% Rh/CoO and 4.8% Rh/CoO, respectively, at 100 °C. (**d**) Product yields of 0.2% Rh/CoO over the course of five rounds of successive reaction. Image reproduced from reference [9].

The catalytic performance metrics of the Rh/CoO catalyst are shown below in Figure 7. The researchers found that Rh single atoms exhibited high activity and selectivity for producing linear butyraldehyde from propene, comparable to that of Rh-based homogeneous catalysts. In contrast, the Rh clusters supported on CoO nanosheets showed significantly lower activity, approximately five orders of magnitude lower than that of the single-atom catalysts. Another key finding of the study was the difference in product selectivity—specifically the ratio of linear to iso-butyraldehyde as a function of Rh loading. The authors reported that single Rh atoms were highly selective for the formation of linear butyraldehyde, whereas Rh nanoclusters produced a mixture of linear and branched products. This trend is illustrated in Figure 7.

**Figure 8:**
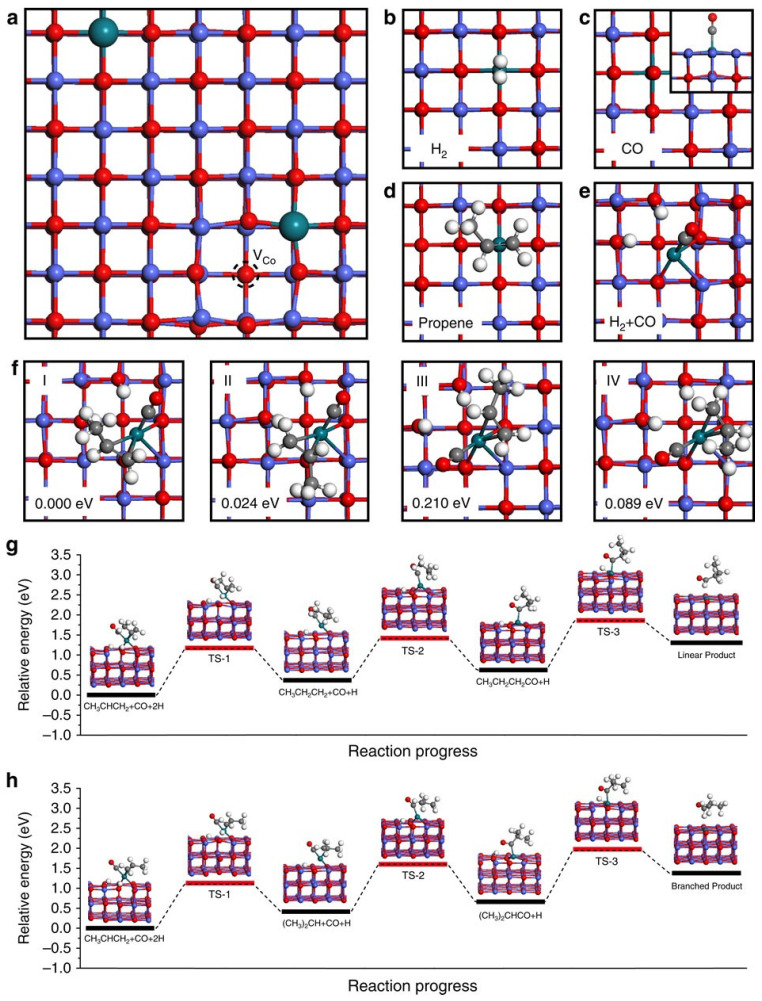
DFT calculations of Rh_1_/CoO in the hydroformylation of propene. (**a**) The model of Rh_1_/CoO showing that Rh atoms (teal) occupy the position of two Co atoms (blue), together with the involvement of a Co vacancy (dotted black circle marked V_Co_. (**b**–**d**) Top views of the adsorption configurations of H_2_, CO, and propene on Rh_1_/CoO, respectively. (**e**) Top view of the co-adsorbed configuration of both H_2_ and CO. (**f**) Four co-adsorbed configurations of reactants and their corresponding adsorption energies. The most stable configuration I was taken as the energy standard (ΔE = 0.0000 eV). Adsorption energies of configuration II, III, and IV were 0.024, 0.210 and 0.089 eV, respectively. (**g**,**h**) Reaction paths deriving from configurations I and II, respectively. Image reproduced from reference [9].

The observed difference in selectivity between single Rh atoms and nanoclusters was further investigated using DFT calculations, as shown in Figure 8. Figure 8a illustrates the adsorption of a single Rh atom, which displaces surface Co atoms, leading to the formation of Co vacancies. Figure 8b–d show the adsorption configurations of individual reactants: H_2_, CO, and C_2_H_4_, respectively. Figure 8e presents the top-view configuration of co-adsorbed H_2_ and CO. The authors noted that co-adsorption of hydrogen and CO on a single Rh atom induces a positional shift in the Rh atom, resulting in a reconstruction of the active site. This structural rearrangement facilitates the adsorption of propene. Figure 8f illustrates the co-adsorbed configurations of the reactants and the catalyst. Among the four proposed configurations, configurations I and II were identified as the most stable based on low adsorption energies, and were subsequently used to calculate the activation barriers and possible reaction pathways, as shown in Figure 8g,h. Based on those calculations, the structure of the hydroformylation mechanism involves the initial addition of a hydrogen atom to one end of the C=C bond in propene, followed by CO insertion at the other end, and finally, the addition of a second hydrogen atom to the carbon in the C=O group. Therefore, the site of initial hydrogen addition plays a crucial role in determining whether linear or branched products are formed.

In the case of Rh nanoclusters, the abundance of available adsorption sites allows hydrogen atoms to attack the propene molecule from multiple directions, resulting in reduced selectivity. This study not only demonstrates an efficient catalyst for hydroformylation with remarkably high selectivity but also provides mechanistic insight into the differences in product selectivity between single-atom catalysts and conventional metal nanocrystals.

**Figure 9:**
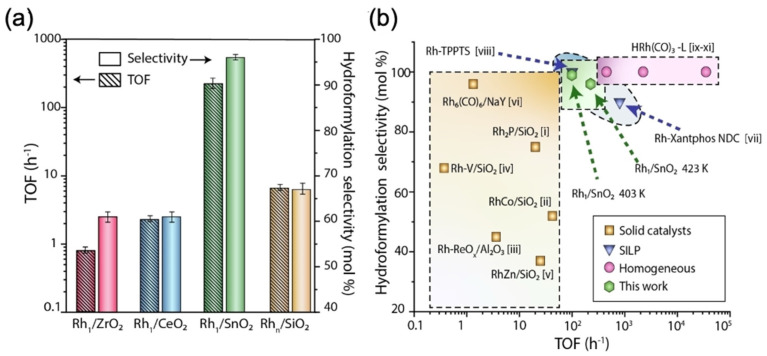
Gas-phase hydroformylation of ethylene. (**a**) Initial ethylene hydroformylation turnover frequency (TOF) and selectivity for single-atom catalysts supported on the series of MO_2_ supports (M = Zr, Ce, Sn) with the performance of a reference Rh_n_/SiO_2_ catalyst included for comparison. Reaction conditions: gas feed Ar:CO:H_2_:C_2_H_4_ = 1/5:1:1:1 (by volume), T = 423 K, P = 20 bar. (**b**) Comparison of the catalytic performance for the Rh_1_/SnO_2_ single-atom catalyst (this work) with homogeneous catalysts (organometallic complexes in solutions) (homogeneous), organometallic complexes in supported ionic liquid-phase (SILP) catalysts, and various all-solid catalysts (solid catalysts). The image was reproduced from reference [7].

Various researchers also explored the effect of surface defects on substrates and how that affects the catalysts’ stability and hydroformylation performance. Recently, Farpón et al. investigated gas-phase ethylene hydroformylation using Rh single atoms stabilized on various oxide supports: ZrO_2_, CeO_2_, and SnO_2_ [7]. Among these systems, Rh single atoms stabilized on SnO_2_ exhibited exceptional catalytic performance and selectivity, comparable to that of organometallic molecular catalysis in liquid media. The catalytic activity followed the order Rh/SnO_2_ > Rh/CeO_2_ > Rh/ZrO_2_. The superior performance of Rh/SnO_2_ was attributed to the reducibility of the SnO_2_ surface during the reaction, creating oxygen-defective sites where the Rh single atoms were stabilized, thus preventing Rh agglomeration. The reductive kinetics of the series of single-atom catalysts scaled in the order Rh/CeO_2_ > Rh/ZrO_2_ > Rh/SnO_2_, indicating an opposite order for the stability of single Rh atoms against reductive agglomeration. The optimal reaction condition temperature for Rh/SnO_2_ was found to be 423 K, with a significant reduction in SnO_2_ and overall catalyst deactivation observed upon further temperature increase (SnO_2_ + 2CO → Sn + 2CO_2_). This study also reported on the hydroformylation of Rh on SiO_2_ support. In this case, the stabilization of Rh single atoms on SiO_2_ was limited by temperature, as a reaction temperature of 423 K resulted in Rh agglomeration to form clusters and nanoparticles. However, Rh/SiO_2_ surfaces exhibited high catalytic activity, albeit lower than Rh/SnO_2_, but substantially higher than Rh/CeO_2_ and Rh/ZrO_2_ surfaces. Figure 9 shows a comparison of hydroformylation activity and selectivity for Rh supported on various oxides, benchmarked against state-of-the-art systems including homogeneous catalysts, organometallic complexes, and other solid catalysts reported in the literature. The data highlights the excellent performance of Rh single atoms supported on SnO_2_.

A number of groups have studied how oxygen deficiencies present in CeO_2_ surfaces can greatly increase the catalytic performance of Rh single-atom catalysts [45,46,47]. Lee et al. [45] deposited Rh onto varying sizes of CeO_x_ clusters supported on Al_2_O_3_. These clusters ranged from 1.08 to 6.20 nm. Rh deposited on all but the largest (6.02 nm) clusters appeared to only form as atomically dispersed species, due to the lack of evidence for Rh-Rh bonds seen in EXAFS spectra. This could be due to the increased number of surface defects present on the smaller ceria clusters, as CeO_x_ defects have been previously observed to stabilize Pt single atoms and anchor them to the surface [48]. Based on combined experimental and theoretical studies, Lee and colleagues believe that the defects allow an increased number of Ce^3+^ to be present on the surface, which modifies the electronic state of the Rh atoms, increasing their electron density and lowering the CO binding strength to a level similar to that of RhCl(PPh_3_)_3_ in a homogenous catalyst [45]. The best catalytic activity of these surfaces was assessed by TOF, which is determined by the moles of butyraldehyde produced during propylene hydroformylation per the moles of Rh per hour. The surface containing 2 wt% Ce, which was composed of Rh atoms deposited on 0.99 nm CeO_x_ clusters, displayed the highest TOF of 16,983 mol_butyraldehyde_ mol^−1^_Rh_ h^−1^ with a conversion of 94%. This reaches the activity of a homogenous Wilkinson’s catalyst, which has a TOF of 15,837 mol_butyraldehyde_ mol^−1^_Rh_ h^−1^ with a conversion of 88% under the same conditions [45].

The same trend was determined by Zheng et al. [46] while investigating the catalytic hydroformylation of propylene over atomically dispersed Rh catalysts present on the (100) facet of CeO_2_ nanocubes calcined at various temperatures. Again, through a combined experimental and theoretical study, they determined that calcination temperatures increased the concentration of oxygen vacancies on the surface of CeO_2_, and calcination at high temperatures modifies the electronic and geometric structures of the Rh species. In situ FTIR spectroscopy and in situ XPS characterizations provide strong evidence that Rh on the 800 °C-calcined 0.06% Rh/CeO_2_ catalyst is easily activated to form surface HRh(CO)_2_ active species for propylene adsorption and activation as well as the subsequent CO insertion [46].

### 3.2. Effect of Synthesis Procedure

The synthesis of the SACs has also garnered attention from the scientific community, as understanding how catalyst synthesis changes the productivity and selectivity of the surface has a major influence on catalytic design. A 2023 review by Tao et al. covered a number of different routes of Rh SAC synthesis such as impregnation [49], metal–ligand coordination [13] and electrostatic adsorption [50] and how the synthesis approach it influenced the TOF for the hydroformylation of various olefins, including ethylene [43]. An example illustrating the impact of synthesis method on catalytic performance is provided below.

**Figure 10:**
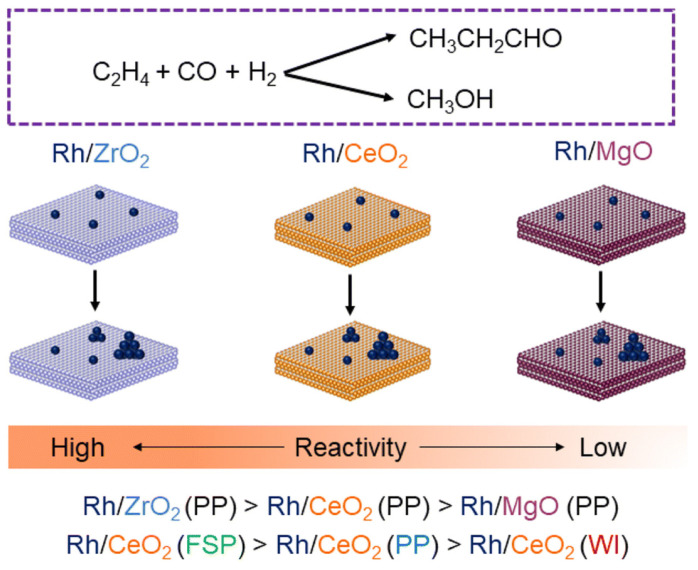
Summary and ranking of reactivity towards the hydroformylation reaction of ethylene with CeO_2_, ZrO_2_, and MgO supported Rh catalysts. The image illustrates that more acidic catalyst supports (ZrO_2_) were shown to be more reactive for ethylene hydroformylation than more basic catalyst supports (MgO). PP, FSP, and WI stand for precipitation, flame spray pyrolysis, and wetness impregnation, respectively. The image isreproduced from reference [24].

In a recent study, Sarma et al. conducted an in-depth investigation into ethylene hydroformylation over Rh single atoms and clusres supported on CeO_2_, MgO, and ZrO_2_ focusing on the influence of synthesis methods on catalytic performance [24]. In this work, the authors used the selectivity toward propanal, the hydroformylation product, as the primary metric to evaluate catalytic efficiency. The authors have explored the effect of four different preparation methods: precipitation, [51] atom trapping, [52,53] pyrolysis of molecular complexes [53] and flame spray pyrolysis (FSP) [54]. These supports are chosen due to their variable acidity, reducibility, and, most importantly, variation in reactivity during the catalytic hydroformylation reaction as reported in the literature [12]. They employed in situ/operando techniques, including diffuse reflectance infrared Fourier transform spectroscopy (DRIFTS) and X-ray absorption spectroscopy (XAS), to analyze catalyst behavior under reaction conditions. Operando spectroscopic analysis revealed the presence of both Rh single atoms and clusters during the reaction. During the reaction, initially, propanal was observed as a hydroformylation product, followed by methanol. The formation of methanol was linked to CO hydrogenation occurring on Rh clusters. As the reaction progressed the Rh single-atoms agglomerate to form clusters leading to a decrease in selectivity for propanal formation and enhanced methanol formation from CO hydrogenation. Comparisons of catalytic activity over various supports showed that acidic supports, such as ZrO_2_, promote the hydroformylation reaction, whereas basic supports, such as MgO, result in negligible catalytic activity. The varying reactivities towards hydroformylation of ethylene is dependent on the preparation method and support, as summarized in Figure 10. Among the catalysts synthesized over CeO_2_ with various methods, catalysts prepared via spray pyrolysis (FSP) showed activity at low temperatures, which indicates that the method of preparation plays a vital role during the reaction.

The observed changes in catalytic reactivity, influenced by both the synthesis method and the choice of support, are largely attributed to the ability to achieve high-surface-area materials by minimizing Rh atom clustering. Surface area plays a critical role in dispersing active sites and varies significantly depending on the support and the preparation method. For example, the authors reported that Rh/CeO_2_ catalysts synthesized via FSP exhibited the highest specific surface area (133 m^2^ g^−1^) compared to those prepared by wet impregnation (56 m^2^ g^−1^) or precipitation (93 m^2^ g^−1^). This enhanced surface area is attributed to the rapid quenching process inherent to FSP, which suppresses particle growth and aggregation, as previously described in the literature [54]. Among the various supports studied (CeO_2_, ZrO_2_, and MgO), ZrO_2_ synthesized via precipitation demonstrated the highest specific surface area (201 m^2^ g^−1^), making it the most active catalyst for the hydroformylation reaction.

### 3.3. Strong Metal Support Interaction in SAC

Since the discovery of strong metal–support interactions (SMSIs) by Tauster et al. in 1978, these interactions have garnered significant attention in the field of heterogeneous catalysis [55]. SMSIs typically manifest through phenomena such as the encapsulation of metal nanoparticles by the support or electron transfer from the support to the metal after high-temperature reduction [56,57,58]. According to the classical SMSI model, platinum nanoparticles tend to become encapsulated by an oxide overlayer under reducing conditions and can be re-exposed upon exposure to an oxidizing atmosphere. Over time, this concept has been expanded to include other forms of SMSI, such as oxidative SMSI (OSMSI) where the support material encapsulates the metal particles under oxidative conditions [59,60,61] and adsorbate-mediated SMSI where adsorbed molecules on a catalyst surface encapsulate metal nanoparticles [62]. SMSIs play multiple critical roles in heterogeneous catalysis. They can stabilize active metal species, enhance metal dispersion, prevent nanoparticle sintering, and improve the accessibility of active sites—ultimately leading to enhanced catalytic activity.

The effect of SMSI in hydroformylation catalysis has been the subject of growing research interest. These interactions introduce a delicate trade-off between catalyst stability, enhanced by encapsulation, and catalytic activity, which can suffer if access to the active site is restricted. Excessive confinement can protect single-atom catalysts (SACs) from deactivation mechanisms such as metal leaching or desorption of metal–ligand complexes, but can also lead to reduced activity due to limited accessibility or high metal coordination environments. This balance is illustrated by several examples from the literature.

In one such study, Amsler et al. investigated Rh single-atom catalysts supported on two oxides: MgO and CeO_2_ [12]. Their results showed that Rh atoms supported on CeO_2_ exhibited higher activity for hydroformylation compared to those on MgO. This was attributed to the stronger confinement of Rh atoms on MgO, which limited their hydroformylation activity compared to those on CeO_2_. The authors provided an in-depth discussion of the hydroformylation mechanism and examined how metal–support interactions influence the stability and reactivity of Rh single-atom catalysts on these oxide supports, including comparisons with homogeneous Rh catalysts.

**Scheme 1:**
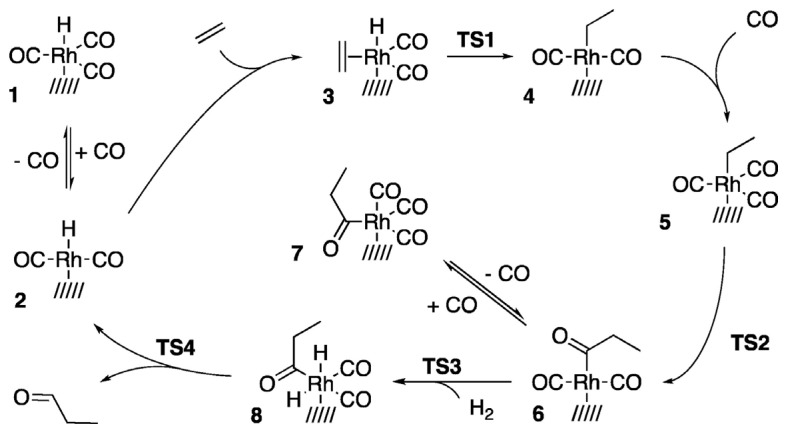
Heck and Breslow mechanism adapted for heterogeneous hydroformylation on supported SACs. Image reproduced from reference [12].

The authors proposed a heterogeneous hydroformylation mechanism (shown in Scheme 1) adapted from the classical Heck and Breslow mechanism for homogeneous systems [63,64]. This modified mechanism consists of the following elementary steps: activation via ligand dissociation (1 → 2), olefin coordination (2 → 3), olefin insertion (3 → 4), CO coordination (4 → 5), CO insertion (5 → 6), oxidative addition of H_2_ (6 → 8), and reductive elimination of the aldehyde product (8 → 2). The cycle involves four transition states (TSs). Within this mechanism, the number of CO ligands coordinated to the metal center in the resting state is likely influenced by the electronic and structural properties of the support.

**Figure 11:**
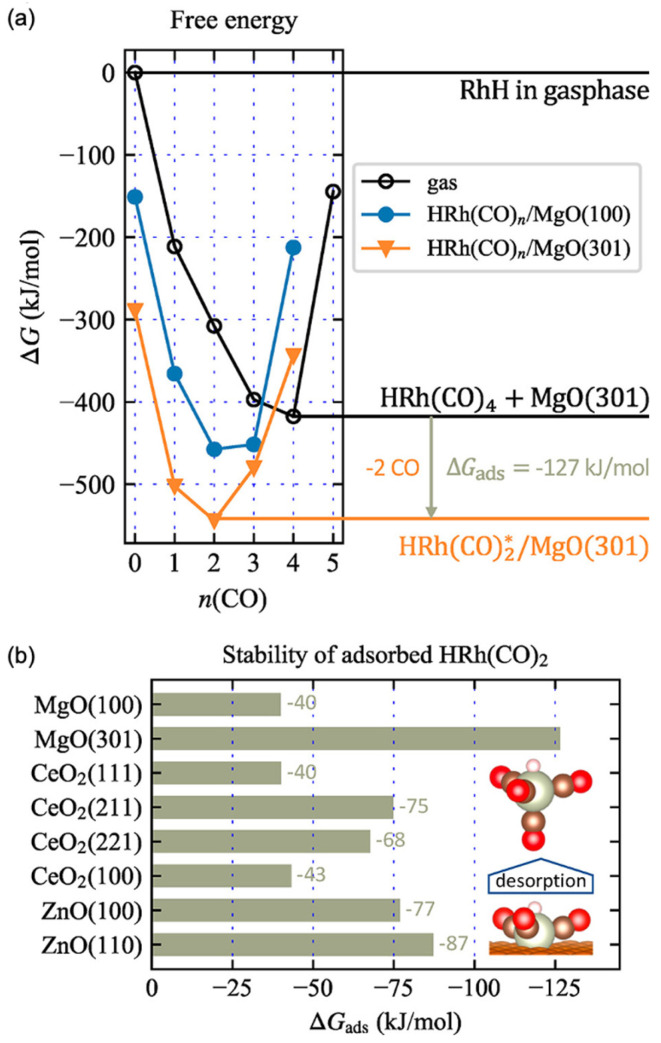
Procedure to obtain adsorption free energies at 363 K and 10 bar: (**a**) Free energy as a function of the number of carbonyl ligands n(CO). (**b**) Adsorption free energies for different SACs. Image reproduced from reference [12].

The authors then investigated the stability of oxide-supported Rh single-atom catalysts using DFT calculations. Desorption of the active rhodium complex into the gas phase or leaching into solution is expected to be a primary deactivation pathway for SACs. To assess catalyst stability, the authors calculated the formation free energy of the supported Rh complexes relative to the most stable gas-phase reference complex, HRh(CO)_4_, as shown in Figure 11. Figure 11a presents the free energies of Rh complexes as a function of the number of CO ligands on two different MgO facets: MgO(100), representing a flat surface, and MgO(301), representing a highly stepped surface. The most stable Rh complex was found on the MgO(301) surface, where Rh binds with two CO ligands. In contrast, on the MgO(100) surface, the lowest-energy configuration involves an Rh complex with three CO ligands. Among the investigated supports, the most stable Rh species was HRh(CO)_2_, which contains two carbonyl ligands. Generally, complexes with three CO ligands are nearly equally stable on flat surfaces with low geometric confinement (i.e., low Rh–O coordination), whereas more open or stepped surfaces such as MgO(301), CeO_2_(211), and CeO_2_(221) tend to destabilize species with more than two CO ligands due to polydentate binding effects. In contrast, flat surfaces like MgO(100) and CeO_2_(111) can better accommodate a third carbonyl ligand. Figure 11b shows a comparison of the stability of the HRh(CO)_2_ complex across various supports. The results indicate that the MgO(301) surface provides the greatest stability for the Rh complex, which the authors attribute to the high confinement effect offered by the stepped surface geometry. This conclusion is further supported by their experimental extended X-ray absorption fine structure (EXAFS) data, which provides structural evidence for the enhanced confinement of Rh species on MgO(301).

**Figure 12:**
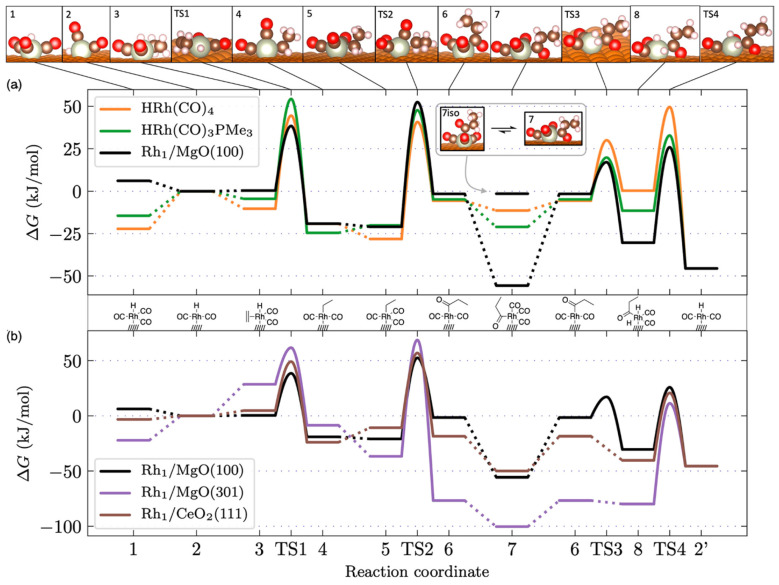
Activity of supported single-atom catalysts (SACs) and molecular catalysts in hydroformylation in gas phase at 363 K and 10 bar: (**a**) Comparison of the supported SAC Rh1/MgO(100) to the two molecular catalysts HRh(CO)_4_ and HRh(CO)_3_PMe_3_. (**b**) Comparison of SACs supported on different metal oxides predicted using differences computed at the PBE-D3 level of theory. Image reproduced from reference [12].

The authors evaluated the catalytic activity of Rh-based systems for ethylene hydroformylation through computational analysis, utilizing reaction energy diagrams shown in Figure 12. Three oxide-supported catalysts were examined: Rh/MgO(100), Rh/MgO(301), and Rh/CeO_2_(111). These were compared with two molecular reference catalysts, HRh(CO)_4_ and HRh(CO)_3_PMe_3_. The resting states for the catalytic cycles were determined based on earlier stability studies as shown in Figure 11. For flat surfaces such as MgO(100) and CeO_2_(111), one CO ligand was removed from HRh(CO)_4_ upon adsorption, leading to the structures illustrated in Scheme 1. In contrast, adsorption on the stepped MgO(301) surface resulted in the removal of two CO ligands from HRh(CO)_4_.

When comparing Rh/MgO(100) to the molecular complexes, the reaction energy barriers were found to be quite similar. A key distinction, however, was the increased stabilization of intermediates 7 and 8 on MgO(100), attributed to a favorable interaction between the carbonyl oxygen of the propionyl group and the support. For both HRh(CO)_4_ and Rh/MgO(100), the highest effective barrier occurred at TS4, corresponding to the hydrogenolysis step, with activation energies of 78 kJ/mol and 82 kJ/mol, respectively. In contrast, for HRh(CO)_3_PMe_3_, Rh/CeO_2_(111), and Rh/MgO(301), the highest activation barrier was identified as TS2 (olefin insertion), with barriers of 72, 81, and 123 kJ/mol, respectively. The authors noted that both olefin insertion and hydrogenolysis have been proposed as rate-determining steps, depending on substrate, catalyst, and external conditions such as reactant pressure and substrate temperature.

Overall, the computational results demonstrate that Rh SACs supported on oxides can exhibit catalytic activity comparable to that of molecular catalysts. However, while Rh/MgO(301) shows the greatest thermodynamic stability, it is also the least active. This trade-off is attributed to the strong Rh–support interaction resulting from high confinement at the stepped surface, which enhances stability but restricts the structural flexibility needed for efficient catalysis.

In support of this understanding, a recent study investigated the role of SMSIs in hydroformylation, focusing on Rh single atoms partially embedded on a ZrO_2_ support, further shedding light on this important aspect of catalyst design [44]. The researchers demonstrated that oxidative SMSIs can be effectively utilized in single-atom catalysts (SACs) to enhance both catalytic activity and structural stability under reaction conditions. Upon oxidative treatment, surface-dispersed Rh atoms became partially encapsulated by the ZrO_2_ support. Subsequent reductive activation in H_2_ reversed this encapsulation, restoring the accessibility of Rh active sites.

Under hydroformylation conditions, the partially encapsulated Rh catalyst achieved a turnover frequency (TOF) of 11,097 h^−1^—significantly higher than previously reported Rh-based SACs. In addition to improved catalytic performance, the embedded Rh sites also showed enhanced resistance to metal leaching, attributed to stabilization from the support interaction.

**Figure 13:**
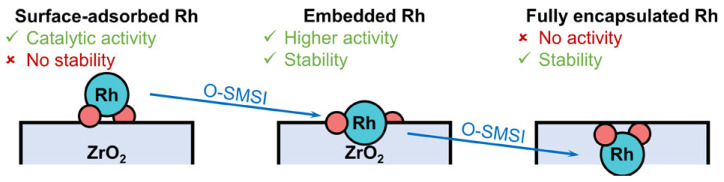
Illustration of how the Rh single atoms embed themselves into the ZrO_2_ support, and its effects on the activity and stability of the catalyst. Image reproduced from reference [44].

These Rh sites, situated at the surface layer of ZrO_2_ and partially enclosed by the support, exhibit both high activity levels and long-term stability. However, a key challenge remains in precisely tuning oxidative SMSI to maximize the formation of these beneficial embedded configurations while avoiding excessive encapsulation, which could hinder catalytic function as shown in the schematics in Figure 13. This work advances the understanding of oxidative SMSI in the field of single-atom catalysis and provides a foundation for the rational design of next-generation heterogeneous catalysts with improved durability and performance.

## 4. Bifunctional Catalysts

Literature evidence suggests that single-atom catalysts often outperform their nanoparticle or nanocluster counterparts, as discussed above in Section 3. To further enhance catalytic performance, the development of bifunctional catalysts has become an area of growing interest. By incorporating multiple components, these catalysts can facilitate different steps of the reaction pathway, potentially leading to improved activity and selectivity. In fact, many metal-catalyzed reactions are proposed to proceed via bifunctional active sites, where colocalized reactive species enable distinct elementary steps within the catalytic cycle. This bifunctional behavior is well established in homogeneous binuclear organometallic systems [65,66]. Several studies have also demonstrated the superior performance of bifunctional catalysts supported on oxides for hydroformylation reactions, in comparison to monofunctional Rh catalysts on similar supports [67,68]. For example, one of the limitations of oxide-supported metal catalysts in hydroformylation reactions is CO saturation of the Rh active sites, which often results in reduced catalytic activity and poor product selectivity [30]. This issue can be effectively addressed by introducing a second component, as demonstrated in bifunctional catalyst systems [68]. More recently, several studies have explored the synthesis of singly dispersed bifunctional catalysts and investigated how the individual components contribute to different steps in the reaction pathway as shown below by Ro et al. [68,69].

**Figure 14:**
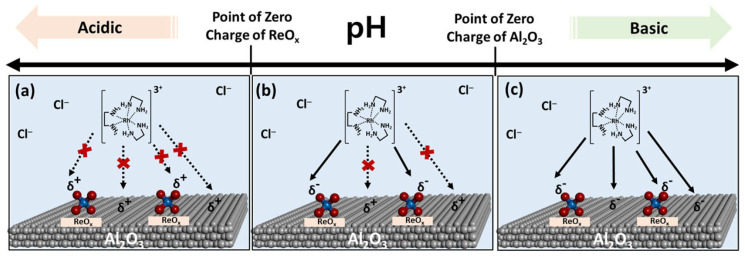
Depiction of how Rh can be selectively deposited near the ReO_x_ particles as opposed to the Al_2_O_3_ support. The figure illustrated deposition at 3 different pHs: (**a**) a pH below the point zero charge (PZC) of both ReO_x_ and Al_2_O_3_ (**b**) a pH above the PZC of ReO_x_ but below the pZC of Al_2_O_3_ and (**c**) a pH above both PZCs. This way, the cationic Rh precursor ([(H_2_NCH_2_CH_2_NH_2_)_3_Rh]^3+^) will only deposit near the negative deprotonated species. Image reproduced from reference [69].

Strong electrostatic adsorption has been shown to be an effective method for the selective deposition of Rh single atoms onto a prepared oxide support. By using pH to control the effective charge of the surface, Ro et al. were able to selectively deposit Rh nanoparticles near atomically dispersed ReO_x_ species on a Al_2_O_3_ support [69]. The catalyst’s synthesis procedure was described as follows: The precursor solution was prepared by dissolving tris-(ethylenediamine)rhodium(III) chloride into 6 mL of water, and the support was suspended in 24 mL of water while being stirred. The pH of both the precursor and the support was adjusted with NH_4_OH or HNO_3_ to an optimal pH of 10 before being mixed together under magnetic stirring. A pH of 10 was chosen as it was above the point of zero charge (PZC) for the ReO_x_ species and near the PZC of the Al_2_O_3_ nanoparticles. This would cause the hydroxyl species on the ReO_x_ to become deprotonated, providing negatively charged oxygens that would attract the cationic Rh precursor while the Al_2_O_3_ nanoparticles would maintain a neutral charge and have minimal interaction with the cationic Rh. This allowed for targeted deposition of the Rh atoms near the ReO_x_ species through the exploitation of site-selective electrostatic interactions, as coulombic interaction is what drives the deposition of the precursor. This process is visualized in Figure 14.

Additionally, the charge state of the Rh species was further tuned by varying the ReOx coverage, which significantly influenced both the reactivity and selectivity of the catalyst toward ethylene hydroformylation by preventing CO saturation of the active sites. This effect arises from the electron-withdrawing nature of ReOx, which renders the Rh species more cationic, thereby weakening the CO–Rh interaction. The reduced CO binding strength in the Rh/ReOx–Al_2_O_3_ system, compared to Rh/Al_2_O_3_, helps prevent CO poisoning of the active sites. Under hydroformylation conditions, the Rh/ReOx–Al_2_O_3_ catalyst exhibits a higher concentration of vacant Rh sites, which are crucial for enhanced activity and selectivity toward propanal formation.

Another example of a bifunctional catalyst is the atomically dispersed rhodium–tungsten oxide (Rh–WO_x_) pair-site catalyst supported on alumina (Al_2_O_3_), as reported by Ro et al. [68]. This system demonstrated increased catalytic selectivity compared to conventional metallic Rh/Al_2_O_3_ catalysts. The improved performance is attributed to the enhanced resistance of Rh–W pairs to CO poisoning during the hydroformylation reaction. In a typical heterogeneous hydroformylation of ethylene over Rh/Al_2_O_3_, CO initially binds to Rh atoms, forming Rh(CO)_2_. For the reaction to proceed, CO must desorb from Rh to allow ethylene adsorption. This desorption step is often problematic due to the strong Rh–CO bond. Rh–W pair sites circumvent this limitation by enabling the transfer of ethylene from tungsten to rhodium without requiring CO desorption, thereby maintaining high catalytic activity and selectivity. At the start of the catalytic cycle, CO binds to Rh while ethylene binds to WO_x_. A dynamic reconfiguration of the Rh–WO_x_ pair site follows, involving the reduction of W^6+^ and formation of the active Rh–W pair. This transformation enables the transfer of ethylene from WO_x_ to Rh. The reaction proceeds through three main steps: (1) reduction of W^6+^ and formation of the active Rh–W site; (2) transfer of adsorbed ethylene from W to Rh without CO desorption; and (3) H_2_ dissociation at the Rh–W interface. Dynamic structural changes in the Rh–W pair during the catalytic cycle are critical for maintaining high activity and selectivity. The authors have noted that the proposed elementary steps resemble those of binuclear homogeneous catalysts, highlighting the mechanistic relevance of atomically dispersed pair sites in bifunctional catalysis [66].

**Figure 15:**
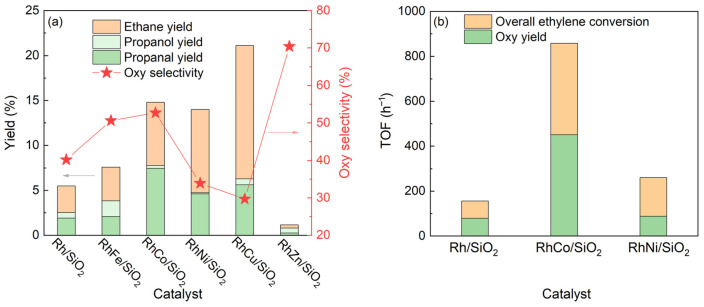
(**a**) Yield, selectivity and (**b**) TOF of various silica supported bimetallic Rh catalysts compared to Rh/SiO_2_. The red trace (**a**) indicates selectivity towards oxy species, or the target products of hydroformylation (propanal and propanol). Catalytic conditions: catalysts exposed to 1:1:1:1 mixture of C_2_H_4_/CO/H_2_/N_2_ at 180 °C after a pretreatment exposure to 25% H_2_/N_2_ at 450 °C for 1 h. Image reproduced from reference [67].

Intermetallic nanoparticles may also enable effective environment control of the active site while maintaining similar site isolation to single-atom catalysts. The catalytic activity of a number of different silica supported bimetallic nanoparticles—including RhFe, RhCo, RhNi, RhCu, and RhZn—were compared using ethylene hydroformylation by Huang et al. (Figure 15a). Nearly all of the bimetallic catalysts, with the exception of the RhZn/SiO_2_ catalyst, outperformed the bare Rh/SiO_2_ catalyst in terms of yields of target products (propanal and propanol). However, the RhZn/SiO_2_ catalysts had the highest selectivity for propanal and propanol, and the selectivity trend follows the sequence RhZn/SiO_2_ > RhCo/SiO_2_ > RhFe/SiO_2_ > Rh/SiO_2_ > RhNi/SiO_2_ > RhCu/SiO_2_ [67]. RhCo/SiO_2_ proved to be the best catalyst in that it maintained both a higher yield and selectivity of targeted products. When comparing the TOF of the RhCo/SiO_2_ to that of bare Rh/SiO_2_, the RhCo/SiO_2_ catalyst had a TOF towards propanal and propanol of ~450 h^−1^, almost five times higher than that of the bare Rh/SiO_2_ catalyst [67].

The study demonstrates that both catalytic activity and selectivity can be modulated by carefully selecting the bimetallic system. While the RhCu/SiO_2_ catalyst showed the highest overall yield of products, it proved to have the lowest selectivity towards propanal and propanol and instead had high selectivity for ethene hydrogenation into ethane. This may be due to phase separation of the RhCu nanoparticles, as the solubility of Cu in Rh is limited below 1100 °C, which may result in the formation of Rh-doped Cu particles (instead of alloyed nanoparticles) that are more favorable for catalyzing alkene hydrogenation [67,70]. In contrast to the RhCu/SiO_2_ catalyst, the RhZn/SiO_2_ catalyst has the lowest overall yield of products but the highest selectivity towards propanal and propanol. Huang et al. believes that this may be due to the Rh surface being covered by unreduced ZnO, as the feasibility of forming an alloy catalyst with Rh is quite low and the RhZn particles failed to show high intensity of either linear or gem-dicarbonyl CO binding in CO-DRIFTS experiments [67]. This tunability of reactivity and selectivity by choosing specific bimetallic catalysts shows promise in creating extremely variable, tunable hydroformylation catalysts.

## 5. Conclusions and Perspectives

In this article, we summarize recent advancements in heterogeneous hydroformylation catalysis involving Rh supported on metal/oxide interfaces. The first section of the paper discusses the influence of Rh particle size and structural characteristics on hydroformylation performance, primarily based on surface science model studies [11]. The optimal size of Rh nanoparticles is reported to be approximately 2.5 nm on SiO_2_ supports, as this dimension maximizes the number of edge and corner sites that promote CO insertion, as demonstrated by McClure et al. through model investigations [11]. Despite these insights, comprehensive surface science studies examining the structure, morphology, and electronic states of Rh clusters on alternative oxide substrates remain scarce. Addressing this gap requires increased attention from the surface science community to enable further progress in this area.

It is well established in the literature that single atoms and atomically dispersed particles generally outperform nanoparticles in terms of catalytic performance, as discussed in Section 3 and Section 4. However, the design and stabilization of single-atom catalysts (SACs) remain significant challenges. A common strategy for synthesizing SACs involves employing low metal loadings to maximize the dispersion of isolated atoms on the surface. Nevertheless, under reaction conditions, these single atoms often tend to aggregate into clusters, leading to a loss of selectivity in hydroformylation. One promising approach to enhance the stability of SACs is the intentional creation of surface defects that can serve as anchoring sites for single atoms. Stabilizing Rh single atoms at defect sites on SnO_2_ and CeO_2_ surfaces has been demonstrated to significantly improve hydroformylation activity and selectivity, in contrast to oxide supports without defect sites [7,45,46,47]. This creation of surface defects to control the size and stability of metal clusters has been explored on various carbon supports [71,72]. For example, controlled defect formation on highly oriented pyrolytic graphite (HOPG) via gentle sputtering with inert gases such as argon has been shown to influence the size of Pd nanoclusters [71,72]. On defect-rich surfaces, Pd clusters exhibited smaller sizes and greater resistance to sintering upon annealing at elevated temperatures. A similar strategy could potentially be extended to metal oxide supports; however, this area remains underexplored. Further research, particularly surface science model studies, is needed to better understand and control defect engineering for the development of high-performance SACs.

Another promising strategy in catalyst design involves stabilizing single atoms through ligand functionalization. For example, phosphorus-based ligands coordinated with Rh can replicate the coordination environment of Wilkinson’s catalyst, a well-known system in homogeneous catalysis. Designing ligand-functionalized metal catalysts offers an effective approach to enhancing both the catalytic activity and selectivity of heterogeneous systems. This concept has been successfully applied by the Medlin group, who used phosphonic acids as ligands to functionalize Pd and Pt nanoparticles supported on TiO_2_, enabling control over activity and selectivity in CO_2_ reduction reactions [73,74]. It has been proposed that charge transfer between the ligand and the metal center modulates the electronic properties of the active site. The spatial arrangement and tunable electronic characteristics of the ligand–metal interface influence the binding strength of reactants and products, while also stabilizing key reaction intermediates. In this way, the combined effects of the metal center and the ligand govern both the activity and selectivity of a catalytic process.

Recent work by the Medlin group has shown that ligand functionalization of Rh single atoms supported on TiO_2_ can significantly enhance hydroformylation performance. This improvement is attributed to changes in the local electronic environment of the Rh active sites induced by the presence of ligands [75]. In addition to modifying catalytic activity, ligand functionalization has also been observed to assist in the formation of atomically dispersed metal species on catalyst surfaces. For instance, Chen et al. developed a metal–ligand self-assembly strategy to generate atomically dispersed Ir atoms on CeO_2_ and MgO supports using 1,10-phenanthroline-5,6-dione (PDO) or 3,6-di-2-pyridyl-1,2,4,5-tetrazine (DPTZ) as ligands. They demonstrated that this approach produced highly uniform, cationic Ir single atoms that remained stable and resisted aggregation even after prolonged reaction times during ethylene hydrogenation [76]. These findings support the idea that functionalizing catalysts with organic molecules possessing tunable charge distributions and electronic properties can provide a general and versatile strategy for heterogeneous catalyst design, mirroring the role of ligands in molecular catalysis. Continued research in this direction could play a critical role in advancing the rational design of efficient catalysts for hydroformylation and other important chemical transformations.

The use of bifunctional catalysts represents a powerful strategy for the design of novel catalytic systems, as the presence of multiple active components can facilitate different steps of a reaction, potentially enhancing both activity and selectivity. As discussed in Section 4, the incorporation of ReO_x_ or WO_x_ species alongside Rh sites has been shown to improve catalytic performance compared to Rh supported on Al_2_O_3_ alone [68,69]. In both cases, the enhanced activity and selectivity are attributed to the increased resistance of the bifunctional sites to CO poisoning, relative to monofunctional Rh sites. The rational design of such bifunctional systems, along with the development of surface science model studies to investigate their structure and function at the atomic scale, will be critical for advancing this area of catalysis.

Computational studies play a crucial role in understanding the atomistic details of catalytic processes and in guiding the rational design of improved catalysts through predictive screening. However, atomic-level information and theoretical modeling in this area remain limited. Ghoshal et al. made significant progress by combining experimental and theoretical approaches, conducting a comprehensive screening of various metal nanoclusters on SiO_2_ supports, as summarized in Section 2 [32]. Their calculations provided important insights into the roles played by different sites on the nanoclusters: edge and corner sites were found to be more selective for hydroformylation, while flat surfaces favored hydrogenation. Furthermore, Ghoshal et al. demonstrated why Rh is a superior catalyst compared to other metals. This is attributed to the optimal binding strength of the CO–Rh interaction. In contrast, Pd binds CO weakly because of Palladium hydride formation, while Pt and Ir bind CO too strongly, leading to higher activation barriers for the CO–C_2_H_5_ coupling steps. It is worth noting, however, that the nanoclusters in their study were modeled as isolated entities, rather than being explicitly supported on SiO_2_. As a result, the effects of the support and the metal–support interface on reactant binding were not fully captured. Incorporating these factors in future studies could provide a more comprehensive understanding of catalytic behavior. To gain a more complete understanding, further model studies that include substrate effects—both experimentally and computationally—are necessary. 

In recent years, the integration of machine learning (ML) into various research areas has proven to be a powerful tool to build a large database which can then be used for experimental design. Applying ML to develop theoretical models that assess the effects of Rh cluster size and shape on hydroformylation performance offers a promising approach to tuning catalytic activity and enabling the rational design of new catalysts. There are already examples in the literature, such as the work by Manna et al., who developed a database of various metal clusters of various sizes with geometries optimized for minimum energy [77]. Expanding this type of computational work to incorporate substrate effects—by modeling how these nanoclusters interact with different supports—combined with ML can effectively guide experimental efforts in the design and optimization of next-generation catalytic materials.

The use of AI/ML-based approaches in guiding catalyst design and process development has proven highly valuable across various research domains. For example, Guan et al. investigated the hydrogen evolution reaction by proposing a novel AI/ML-based screening strategy to identify perovskite catalysts that are more efficient and less energy-intensive [78,79]. Their approach introduces “A-site ionic electronegativity (AIE)” as a key descriptor for evaluating catalyst performance in the hydrogen evolution reaction. The AIE descriptor captures the interactions between A-site (larger-radius cationic metals) and B-site (smaller-radius cationic metals) elements within the perovskite structure. This approach has demonstrated significantly improved predictive power compared to traditional screening methods that focus solely on A-site properties [73]. By using AIE as a guiding parameter, this screening strategy reduces both the time and cost required to develop high-efficiency perovskite catalysts for hydrogen production, ultimately enhancing the overall efficiency of catalyst design.

Finally, while rhodium remains the most widely used metal for industrial hydroformylation due to its high activity and selectivity, it is expensive, rare, and increasingly scarce. Consequently, the search for alternative, earth-abundant catalysts is a growing priority in sustainable catalysis [80]. Among potential alternatives, cobalt has shown the most promise in approaching rhodium-like performance. However, progress with other metals has been limited by a lack of detailed mechanistic understanding, particularly concerning the rate-limiting steps. Future efforts focused on ligand design, mechanistic elucidation, and the development of suitable non-rhodium precursors may help bridge this gap and expand the scope of sustainable hydroformylation catalysis.

## Data Availability

No new data were created or analyzed in this study.
